# Case report: Successful treatment of intestinal leiomyositis in a dog using adjunctive intravenous immunoglobulin

**DOI:** 10.3389/fvets.2024.1373882

**Published:** 2024-07-23

**Authors:** Michelle Patrick Olivarez, Jarod Williams, Nutnapong Udomteerasuwat, Sarah Corner, Christopher Byers

**Affiliations:** ^1^Southeast Veterinary Referral Center, Miami, FL, United States; ^2^Ozark Veterinary Specialty Care, Springdale, AR, United States; ^3^Veterinary Diagnostic Laboratory, Michigan State University, East Lansing, MI, United States; ^4^CriticalCareDVM.com, Omaha, NE, United States

**Keywords:** intestinal leiomyositis, ileus, pseudo-obstruction, CIPO, intravenous immunoglobulin (IVIg), cholecystitis, T-cell activation

## Abstract

A 10-year-old spayed female Dachshund presented with abdominal pain and generalized severe ileus. An exploratory laparotomy was performed, confirming a severe ileus of undetermined origin. Multiple intestinal biopsy results confirmed acute intestinal leiomyositis. Immunohistochemistry (IHC) stains confirmed a T-cell predominant inflammatory infiltrate. Intravenous immunoglobulin (hIVIG) was administered prior to immunosuppressive therapy. Within 10 days of hIVIG treatment, functional peristaltic activity returned, and symptoms resolved. Long-term management, including the use of mycophenolate, resulted in sustained functional peristaltic recovery. Further studies are needed to explore the potential benefits of hIVIG treatment in the stabilization phase of this commonly fatal, treatment-refractory disease.

## Introduction

This case report describes the successful treatment of pseudo-obstructive intestinal leiomyositis in a dog utilizing conventional therapy with adjunctive intravenous immunoglobulin. Immunopathology performed documents a T-cell predominant myo-inflammatory infiltrate. To our knowledge, functional peristaltic long-term recovery has not been previously documented in a dog.

## Case description

A 10-year-old spayed female Dachshund weighing 5.5 kg was presented to her primary care veterinarian for evaluation of weight loss, anorexia, vomiting, and lethargy for several days’ duration. Physical examination abnormalities included mild sinus tachycardia (heart rate 160 bpm), weight loss (body weight 5.7 kg; 1.8 kg loss over 1 month), depression, and mild dehydration. Point of care complete blood count (CBC), biochemical profile (CHEM), and urinalysis (UA) were performed (IDEXX^1^). Biochemical abnormalities included hyperglycemia 240 mg/dL (70–143 mg/dL), BUN elevation 44 mg/dL (7–27 mg/dL), normal creatinine 0.5 mg/dL (0.5–1.8 mg/dL), hyponatremia 110 mmol/L (144–160 mmoL/L), hypochloridemia 68 mmol/L (109–122 mmoL/L), and hypokalemia 2.7 mmol/L (3.5–5.8 mmoL/L). Urinalysis collected by cystocentesis revealed isosthenuria 1.014 (1.015–1.045) without proteinuria or glucosuria. Supportive care was initiated, including intravenous (IV) fluids (Normosol-R); an initial 10 mL/kg IV bolus followed by 10 mL/kg/h IV; potassium chloride (20 mEq/L); and maropitant (1 mg/kg IV). The patient was subsequently referred for continued care and further diagnostic investigation.

Upon arrival at the Referral Center Emergency Service (AESC^2^), the vital signs were normal, and Doppler blood pressure was measured at 156 mmHg (reference range: 90–140 mmHg). Point-of-care testing showed a packed cell volume (PCV) of 57% and total solids (TS) of 7.0 g/dL (reference ranges: 42–54% and 5.9–7.8 g/mL, respectively). C-reactive protein was elevated at 8.6 mg/dL (references: < 1.0 mg/dL), baseline cortisol was >10.0 (reference: 2.0–9.0 ug/dL); and symmetric dimethylarginine (SDMA) was at the upper limit of normal at 14 (reference: 0–14 ug/dL). Venous blood gas analysis showed the following: pH 7.59 (reference: 7.35–7.43), HCO3 46.4 mmol/L (reference: 22.2–22.4 mmol/L), PCO2 52.0 mmHg (reference: 29–42 mmHg), tCO2 48.0 mmol/L, sodium 128 mmol/L (reference: 146–154 mmol/L), potassium 2.3 mmol/L (reference: 3.8–5.3 mmol/L), and chloride 85 mmol/L (reference: 105–115 mmol/L), cumulatively consistent with hypochloremic metabolic alkalosis.

**Figure 1:**
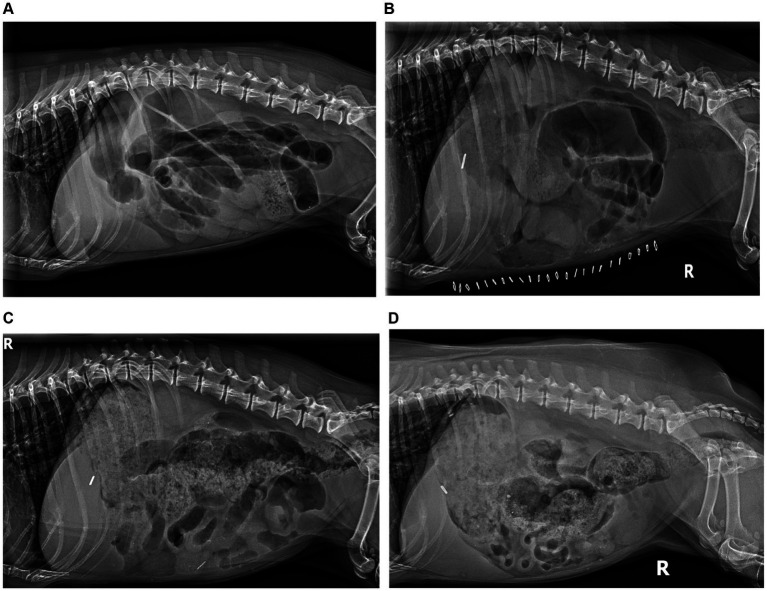
Radiographic peristaltic recovery. **(A)** Day 0 RLAT: severe generalized gaseous ileus, segmental luminal fluid, and luminal density consistent with functional and physical obstruction. **(B)** Day 7 RLAT: severe ileus persists with variable segmental fluid distension; no visible ingesta, consistent with ongoing hyporexia. **(C)** Day 15 RLAT: significant ingesta is present, colonic gaseous distension with formed fecal matter, significantly diminished small bowel distension, and peristaltic contractions are evident. **(D)** Day 111 RLAT: normal gastric ingesta, formed stool present in the colon, resolved small bowel distension with frequent segmental peristaltic contractions evident.

Abdominal radiography revealed generalized dilation of the gastrointestinal tract with no radiopaque foreign body (Figure 1). Thoracic radiography was unremarkable. Supportive care was continued, including intravenous lactated ringers solution (30 mL/h) with KCl supplementation (20 mEq/L), maropitant (1 mg/kg IV q24h), and ampicillin/sulbactam (20–22 mg/kg IV q8h).

Comprehensive abdominal ultrasonography was performed by a board-certified veterinary small animal internal medicine specialist (OVSC^3^) and revealed severe, generalized ileus of the small intestine and stomach. The bowel luminal content was reported as admixed fluid and gas without coordinated propulsion. Additional findings included mild mesenteric lymphadenomegaly, moderate-to-marked gall bladder distension with heterogeneous dynamic inspissated bile, mild heterogenous hepatic parenchyma, and bilateral prominent adrenal glands. Due to the severe ileus, a positive contrast gastrointestinal (GI) study was not performed, and an exploratory laparotomy was recommended. Premedication included lidocaine 2% (2 mg/kg IV), midazolam (0.25 mg/kg IV), and hydromorphone (0.1 mg/kg IV). General anesthesia was induced with propofol (4 mg/kg IV) and maintained with isoflurane while providing mechanical ventilation (ADS2000^4^).

A ventral midline celiotomy was performed. The stomach and small intestine were found to be severely distended and atonic. A plug of compressible ingesta was palpable in the distal ileum and successfully digitally advanced antegrade through the ileocecocolic valve; this plug was considered insufficient to primarily obstruct the GI tract. Incisional biopsies were obtained from the stomach, duodenum, jejunum, and ileum using a standard incisional technique. The liver appeared normal in color and size; a wedge biopsy was collected from the right lateral lobe. The gall bladder was moderately distended with palpably granular material, necessitating a cholecystectomy. Additionally, colonic distension and atony were observed, and the adrenal glands were prominent and nodular. Recovery from anesthesia and surgery was uneventful.^5^^,^^6^

Postoperative care included dexamethasone sodium phosphate (0.1 mg/kg IV once), cobalamin (500 mcg SC once), vitamin B complex (0.5 mL SC once), ampicillin/sulbactam (22 mg/kg IV 8 h), enrofloxacin (10 mg/kg IV q24h), lidocaine (25 mcg/kg/min IV CRI), hydromorphone (.013 mg/kg/h IV CRI), metoclopramide (1 mg/kg/day IV CRI), 0.9% NaCl (120 mL/kg/day IV CRI x 18 h), and KCl (30 mEq/L). As the patient tolerated alimentation, IV fluid support was gradually weaned, and the patient was transitioned to oral medication equivalents, including prednisolone (0.46 mg/kg PO Q24h), enrofloxacin (11.7 mg/kg PO q24h), metronidazole (22 mg/kg PO q12h), ursodiol (13 mg/kg PO q24h), metoclopramide (0.43 mg/kg PO q12h), tramadol (4.3 mg/kg PO q6h), and gabapentin (17 mg/kg PO 12 h). Tramadol was chosen over a pure mu agonist to avoid opioid-exacerbated ileus. Daily laboratory monitoring revealed normalization of electrolyte deficiencies by 72 h postoperatively. A commercial prescription low-fat diet (Hills I/D LF^7^) was prescribed postoperatively and continued during the recovery period.

Histopathology of the duodenum, jejunum, and ileum revealed severe, diffuse, chronic lymphoplasmacytic-to-neutrophilic leiomyositis of the tunica muscularis, as well as moderate lymphoplasmacytic enteritis with luminal bacteria and protozoa (suspected ciliates). In all sections of the small intestine, the tunica muscularis was infiltrated by moderate numbers of lymphocytes, plasma cells, and neutrophils. The smooth muscle within the affected areas was severely disrupted, with smooth myocytes variably vacuolated, fragmented, or replaced by plump fibroblasts amid fine collagen fibers, resulting in the loss of the muscular architecture of the circular longitudinal muscle layers. Neurons within the myenteric plexus within the tunica muscularis remained intact. The villous lamina propria contained moderate numbers of lymphocytes and plasma cells, as well as fewer eosinophils and neutrophils (Supplementary Figure S1). Immunohistochemical staining of the small intestine for lymphocyte markers CD3 and CD21 showed mixed lymphocytic and neutrophilic inflammation with T-cell predominance (Supplementary Figure S2). The stomach was within normal limits. The gall bladder lamina propria was diffusely expanded by moderate numbers of neutrophils, lymphocytes, and plasma cells, consistent with moderate suppurative and lymphoplasmacytic cholecystitis; segmental cystic mucinous hyperplasia was also present. Liver histopathology revealed mild centrilobular hepatocellular vacuolation. Bile aerobic bacterial culture grew *Escherichia coli* and *Enterococcus hirae,* susceptible to enrofloxacin and amoxicillin/clavulanate, respectively. Serum cobalamin was normal at 697 ng/L (251–908 ng/L), and mild hypofolatemia at 6 (7.7–24.4 ug/L) was documented. The vitamin D profile revealed an elevated parathyroid hormone at 24.8 (1.1–10.6 pmol/L), mild ionized hypocalcemia at 1.15 (1.25–1.45 mmoL/L), and normal 25-hydroxyvitamin D at 239 (109–423 nmol/L), most consistent with secondary hyperparathyroidism.

The patient was evaluated 1 week postoperatively. The primary symptoms reported were inappetence without vomiting, regurgitation, or diarrhea. Point-of-care CBC showed mild neutrophilia (13,410/uL) and mild eosinopenia (10/uL), while CHEM and electrolyte profiles were normal [IDEXX (see text footnote 1)]. A single lateral abdominal radiograph revealed a persistently severe ileus with no evidence of peritoneal effusion. The patient was treated with human intravenous immunoglobulin G [hIVIg; 5 grams IV (1.1 g/kg)] over 6 h (Gamunex^9^). Dexamethasone sodium phosphate (0.1 mg/kg IV) and vitamin B complex (0.5 mL SC) were administered pre-infusion. Dexamethasone was administered to help mitigate potential hIVIG reactions. Amoxicillin/clavulanate (11.5 mg/kg PO q12h) was prescribed for 3 weeks based on bile culture. Multimodal immunomodulatory therapy was also prescribed (prednisolone at 0.46 mg/kg PO q24h for 7 days, then every 48 h; mycophenolate at 9.25 mg/kg PO q12h). Treatment with tramadol, gabapentin, metoclopramide, enrofloxacin, ursodiol, metronidazole, and a low-fat diet were continued as previously prescribed.

The patient was reevaluated 8 days following the hIVIg infusion. The patient’s appetite and thirst had improved, and bowel movements were reportedly normal. Abdominal radiography revealed food in the stomach, consistent with recent meals, and feces in the colon. Small bowel luminal gas was present but markedly improved, with visible peristaltic contractions in the small bowel. Reference laboratory testing (Antech^10^) revealed a mildly elevated ALP (152 U/L), mild thrombocytosis (508,000/uL), and mild neutrophilia (11,760/uL). Continued therapy included Vitamin B complex (0.5 mL SC q7 days x 4 weeks, then q14 days), prednisolone (0.46 mg/kg PO q24h), metoclopramide (0.43 mg/kg PO q12h), and ursodiol (13 mg/kg PO q24h). Mycophenolate had not been started as instructed but was eventually started approximately 4 weeks postoperatively.

At 111 days following hIVIg administration, the patient exhibited normal digestive cycles and weight gain, reaching a body weight of 8.5 kg. Abdominal radiography revealed continued subjective normalization of peristalsis (Figure 1). Metoclopramide was discontinued, and instructions were provided for the gradual weaning of immunomodulatory therapy, starting first with a reduction in prednisolone from 0.29 mg/kg/day to every other day. At the time of writing, more than 2 years postoperatively, the patient remains in functional remission and taking mycophenolate every other day (approximately 5.8 mg/kg, weight gain adjusted) and ursodiol daily (9.8 mg/kg once daily, adjusted for weight gain) The treatment and progress timeline is provided in Table 1.

**Table 1:** Timeline of treatments and observations.

Timeline	Treatment	Observation
Exploratory laparotomy, day 0	Dexamethasone SP 0.1 mg/kg IV postoperative, fluids, antimicrobials, analgesics, prokinetics, B-12, B-complex, and ursodiol	Radiographic, surgical, and functional severe ileus, 5.5 kg
Surgical discharge day3	Antiemetics, antimicrobials, prednisolone 0.46 mg/kg once daily, bland diet	Marginally improved but not normal appetite, no BM reported, 5.4 kg
Postoperative day 7	Oral medications continued: Dexamethasone SP 0.1 mg/kg, HIVIG 0.6 g/kg infusion, B-complex, prednisolone 0.46 mg/kg/day, mycophenolate prescribed 10 mg/kg orally twice daily, Clavamox extended to 21 days, and analgesics weaned.	Radiographic severe ileus persisted, persistent hyporexia, no fecal eliminations, symptoms, SQF support needed, 5.4 kg
Postoperative day 15	Clavamox, ursodiol, metoclopramide, and prednisolone 0.46 mg/kg	Radiographic peristaltic recovery, functional improvement, normal alimentations and eliminations, 5.4 kg
Postoperative day 30	Mycophenolate started at 10 mg/kg orally twice daily with a gradual reduction, and prednisolone was reduced to 0.29 mg/kg/day	Functional recovery reported by the owner, normal alimentation and eliminations,
Postoperative day 111	Prednisolone was reduced to 0.29 mg/kg every other day and discontinued within 3 months. Mycophenolate was gradually reduced to 10 mg/kg every other day	Radiographic peristaltic recovery, functionally normal alimentation, and eliminations. 8.5 kg
Postoperative day 420	Ursodiol and mycophenolate every other day	Reported normal alimentation and eliminations.

## Discussion

To the authors’ knowledge, this is the first case reporting the use of hIVIg for functional stabilization of intestinal pseudo-obstruction (CIPO) secondary to small intestinal leiomyositis in a dog. The term “intestinal pseudo-obstruction” was introduced in the late 1950s by Dudley et al., who reported 13 human cases of intestinal obstruction unexplained by mechanical origin; this was referred to as “spastic ileus” (1–3). In human medicine, intestinal pseudo-obstruction may be acute or chronic. The acute form has been associated with abdominal surgery, peritonitis, hypokalemia, spinal or pelvic trauma, viral enteritis, myocardial infarction, retroperitoneal hemorrhage, and anticholinergic or opioid treatment (1–5). The chronic form is primarily characterized as congenital or acquired and can be further classified as primary (idiopathic) or secondary disorders (2–5) Acquired forms may occur secondary to neurologic, metabolic, endocrine, paraneoplastic, autoimmune, or infectious etiologies (1–5). CIPO is a rare and highly morbid syndrome that may be considered an insufficiency of the intestinal pump, impairing gastrointestinal propulsion and causing symptoms of functional obstruction without mechanical origin (1, 2, 4–8). Chronic intestinal pseudo-obstruction has been reported in various animal species, including dogs, horses, cats, and birds (4, 6, 9–19).

Affected dogs are presented with non-specific signs, including abdominal pain, nausea, vomiting, regurgitation, bloating, diarrhea, anorexia, abdominal distension, and weight loss of variable onset. Diagnostic imaging reveals marked gastric and small intestinal dilatation with severe hypomotility (4, 7–11, 13–16, 20). In veterinary medicine, clinical and radiologic/ultrasonographic evidence of intestinal obstruction is an indication for exploratory surgery (9). If no evidence of mechanical obstruction is found, full-thickness biopsies should be obtained from each small intestinal segment (9).

Intestinal leiomyositis, characterized by infiltration of the smooth muscle fibers of the tunica muscularis by lymphocytes, is the most frequent CIPO lesion in dogs (6, 9–11, 15, 16). Infiltration of lymphocytes between functional myocytes affects the contractility of the enteric smooth muscle cells, causing subsequent ileus (4, 21). Pathologic features of visceral myopathies reflect degenerative changes, including varying degrees of myofiber atrophy and vacuolar degeneration (22). In the reports of both human and canine patients with visceral myopathies, the predominant inflammatory cell infiltrating atrophic muscle layers are T cells, suggesting a cell-mediated inflammatory reaction directed at smooth muscle cells that can lead to the destruction of the muscularis mucosa (4, 13, 22, 23). This T-cell myopathic-predominant inflammation was identified in the dog presented in this case. With disease progression, smooth muscle is replaced by fibrosis (23). In cases of intestinal leiomyositis, the mucosa, submucosa, and neural plexuses are relatively spared (4, 11, 12, 22). The lack of mucosal lesions makes endoscopic biopsies inadequate to establish a diagnosis (4, 9). The diagnostic yield of conventional, endoscopic superficial mucosal biopsies is low, as submucosal neuromuscular structures are usually missed (4, 24, 25). It is suspected that CIPO is underdiagnosed in dogs, given the lack of full-thickness intestinal biopsies in many dogs with chronic enteropathies and the awareness of this condition among veterinarians (4).

Management of CIPO in people is largely directed at maintaining adequate caloric intake, providing parenteral nutrition, promoting gastrointestinal motility, and treating complications (e.g., bacterial overgrowth, intractable pain) (1, 3, 4, 7). Prokinetic treatment is a mainstay of treatment. Cisapride has been shown to increase the antroduodenal motility index and improve enteral feeding in people; it has also been shown to increase lower esophageal sphincter pressure and decrease gastric reflux in dogs (4, 25–28). In dogs, cisapride is a more potent and effective prokinetic agent compared to metoclopramide (1, 26). Other drugs, such as erythromycin, azithromycin, or mitemcinal (an erythromycin-derived motilin agonist), have been shown to stimulate antral motility in humans and dogs with functional or experimentally-induced gastric obstructions (4, 29, 30). However, to date, none have been shown to reliably improve gastrointestinal function in dogs with leiomyositis (4, 19).

Immunomodulatory agents, including corticosteroids, cyclosporine, cyclophosphamide, and azathioprine, have been used in humans and canine patients with documented intestinal leiomyositis (3, 4, 12, 20, 23, 31, 32). Such therapy is thought to be most successful when initiated early in the course of the disease, before mural muscular atrophy and fibrosis develop (3, 4, 12, 31, 32). Despite treatment, the overall prognosis for both human and canine patients remains poor (4).

Statistically justified pathology and treatment-specific guidelines for intestinal leiomyositis are not available in canine species. Considering the cumulative case knowledge and outcomes reported in veterinary databases, commonly used antimicrobial, antiemetic, antisecretory, and analgesic therapies may have reasonable application but do not appear to affect outcomes significantly, with average survival periods often reported in single-digit weeks (4, 6, 19, 33, 34). In cases that have employed immunosuppressive therapy, outcomes remain poor overall when the pathophysiologic component of mural myofiber atrophy or fibrosis is observed (4, 6, 9, 13, 15). However, treatment success with a functional outcome was reported in a single dog using combined immunosuppressive therapy with prednisolone and azathioprine before the development of mural muscular atrophy and fibrosis (32).

A comprehensive review of cumulative pathologic findings relative to treatment responses may be helpful but is beyond the scope of this report. A brief overview is provided (Table 2).

**Table 2:** Summary of reported histopathology, treatment(s), and survival of canine intestinal leiomyositis/chronic intestinal pseudo-obstruction.

Report	Pathology	Treatment	Survival
6-dog retrospective, 4	Variable intestinal leiomyositis, CD3 predominant, variable-consistent tunica muscularis atrophy, variable fibrosis, and variable enteric plexus neuritis	Combined antiemetic, prokinetic, antimicrobial, immunomodulatory, and treatment agents	No functional peristaltic recovery; average survival of 19 days (3–270)
1 yo F Bernese mountain dog, 6	Intestinal leiomyositis, tunica muscularis atrophy, fibrosis, neuronal atrophy, ganglioneuritis	Combined antiemetic, prokinetic, antibiotic, and immunosuppressive (prednisone) treatments	No functional peristaltic recovery; survival of 14 days
2 yo M English Bulldog, 9	Intestinal leiomyositis, tunica muscularis atrophy, and fibrosis	Combined antiemetic, prokinetic, antimicrobial, and immunosuppressive (prednisone and azathioprine)	No functional peristaltic recovery; survival of 2 weeks
4 yo FS Bernese mountain dog, 13	Intestinal leiomyositis, fibrosis, CD3 predominant, tunica muscularis atrophy, intact myenteric plexi	Combined antiemetic, prokinetic, antimicrobial, and immunosuppressive (prednisolone)	No functional peristaltic recovery; survival of 17-days
3 yo MN border collie, 15	Intestinal leiomyositis, tunica muscularis atrophy, and fibrosis	Combined antimicrobial and immunosuppressive (prednisone) treatment	No functional peristaltic recovery or survival
7 yo MN mixed canine 60	Intestinal leiomyositis, CD3 predominant, intact muscularis, no fibrosis,	Initially, antimicrobials, prokinetics, analgesics, then immunosuppressive prednisone and azathioprine were added on day 59, and medications weaned	No radiographic assessment, but functional recovery reported on day 270 after discontinuing medication
10 yo FS daschund	Intestinal leiomyositis, CD3 predominant, intact muscularis, no fibrosis	Antiemetics, antimicrobials, prokinetics, analgesics, antiinflammatory prednisolone, HIVIG, and mycophenolate	Full radiographic and functional recovery of >2.5 yrs

As the predominant inflammatory cell in intestinal leiomyositis is the T cell, hIVIg represents a reasonable therapeutic option. Treatment with hIVIg was chosen due to the historically poor prognosis associated with standard multimodal immunomodulatory and prokinetic therapy for the treatment of intestinal leiomyositis. Given the phase of surgical healing, conventional immunosuppression could introduce significant recovery risk without predictable benefits. Human IVIg is not suspected to negatively affect tissue healing and is used perioperatively in humans with specific conditions (35). Specific effects of hIVIg on tissue healing are not well described. hIVIg does not appear to provide long-term immune-suppressive benefits (36).

Human intravenous immunoglobulin (hIVIg) is composed of highly purified immunoglobulin G, obtained from large pools of donated human plasma, and has been used for more than 45 years to treat a variety of diseases in both humans and dogs (36–42) (Table 3). Its mechanism of action is complex and includes modulation of expression and function of Fc receptors, interference with the activation of B and T cells and complement, and a decrease in immunoglobulin production (37, 38). Disorders that have reportedly responded to hIVIG include a wide spectrum of diseases mediated by autoantibodies or believed to depend primarily on autoaggressive T cells; hIVIg is a component of therapy used for categorical autoimmune gastrointestinal motility disorders in humans, under which leiomyositis broadly falls (38, 54).

**Table 3:** Indication and efficacy of HIVIG therapy in dogs (43).

Indication	Outcome
IMHA (immune-mediated hemolytic anemia)	HIVIG treatment in combination with conventional immune-suppressive therapy (CIST) was based on three studies; the cost–benefit was not justified (37, 44, 45).
ITP (immune-mediated thrombocytopenia)	HIVIG treatment, in combination with CIST, expedites platelet recovery compared to CIST therapy alone (41). HIVIG treatment showed improved platelet recovery comparable to Vincristine with CIST, but the cost–benefit was not justified (46). HIVIG treatment with paraneoplastic ITP appeared to be safe but without benefits (47).
Evans syndrome	HIVIG treatment, in combination with leflunomide in a diabetic dog, showed rapidly improved platelet but not erythrocyte counts (48).
Cutaneous autoimmune disease	HIVIG treatment showed potential benefits in the treatment of trimethoprim sulfa associated with Stevens-Johnson syndrome (49) and drug eruption necrotic dermatitis (50). Single-case report of benefit in combination with CIST for pemphigus foliaceous management (51).
Myasthenia gravis	HIVIG treatment showed uncertain benefits demonstrated in the two dogs treated in combination with conventional myasthenic therapies, and the cost–benefit was not justified (52).
SARDS (sudden acquired retinal degeneration syndrome)	HIVIG treatment showed no identifiable vision salvage benefits in an 8-dog series, and the cost–benefit was not justified (53).

Treatment with hIVIg has not been previously reported in a veterinary patient with intestinal leiomyositis. This patient’s median survival time far exceeded those reported in previous publications (4, 11, 15). Serial radiography well documents functional peristaltic recovery that was not previously reported. This patient was treated with hIVIg 1 week postoperatively, and we propose that early intervention positively contributed to our patient’s response.

Dexamethasone sodium phosphate, administered immediately and 1 week postoperatively, would not have a predictable long-term immunomodulatory effect. Prednisolone therapy was initiated at an antiinflammatory dose after treatment with hIVIg. Mycophenolate therapy was prescribed to control T-cell-initiated immunopathology but was not started until after 1 month postoperatively. Clinical and radiographic improvements were temporally correlated with hIVIG therapy but cannot be verified by this single case.

It is unclear if cholecystectomy and the resolution of bacterial cholecystitis provided a long-term impact on this patient’s response to the prescribed therapies. A recent report by Viljoen et al. documented a correlation between inflammatory enteropathies and cholecystitis (55). In human patients, infectious molecular mimicry such as prodromal enteritis has been reported (31, 56). To our knowledge, there is no known association between cholecystitis and leiomyositis in dogs.

The functional long-term response to therapy, including hIVIg in this patient, warrants further investigation as a treatment option during the stabilization phase of intestinal leiomyositis in dogs.

## Data availability statement

The original contributions presented in the study are included in the article/Supplementary material, further inquiries can be directed to the corresponding authors.

## Ethics statement

The animal studies were approved by OVSC Internal Medicine Clinical Ethics Committee. The studies were conducted in accordance with the local legislation and institutional requirements. Written informed consent was obtained from the owners for the participation of their animals in this study.

## Author contributions

MO: Writing – original draft, Writing – review & editing. JW: Writing – review & editing. NU: Writing – review & editing. SC: Writing – review & editing. CB: Writing – review & editing.

## Footnotes

^1^IDEXX Laboratories, Inc., Westbrook, Maine 04092.

^2^Animal Emergency and Specialty Center, Springdale, AR 72762.

^3^Ozark Veterinary Specialty Care, Springdale, AR 72762.

^4^A.D.S. 2000, Engler Engineering Corporation, Hialeah, FLA 33013.

^5^Michigan State University, Veterinary Diagnostic Lab, Lansing, MI 48910.

^6^Texas A&M University, Gastrointestinal Lab, College Station, TX 77843.

^7^Hills Pet Nutrition, Inc., Topeka, KS 66601.

^9^Gamunex-C, Grifols Inc., Research Triangle Park, NC 27709.

^10^Antech Diagnostics, Inc., Fountain Valley, CA 92728.
