# Supplementary figures and images for: Case report: Successful treatment of intestinal leiomyositis in a dog using adjunctive intravenous immunoglobulin
Source: Front Vet Sci. 2024 Jul 23;11:1373882. doi: 10.3389/fvets.2024.1373882 (PMC11300369; doi:10.3389/fvets.2024.1373882)

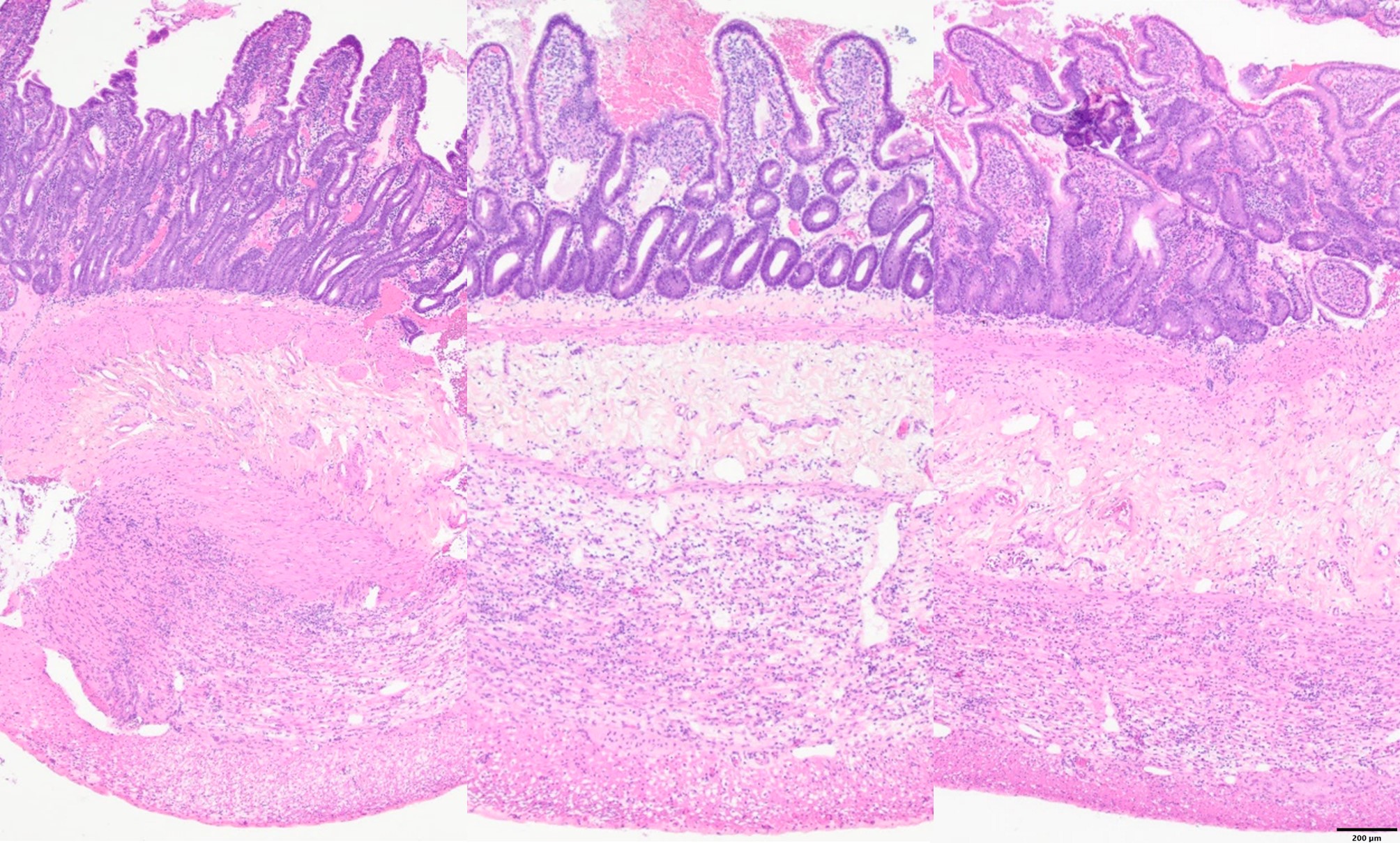

Supplement: Supplementary file 1 [file Image_1.jpeg]

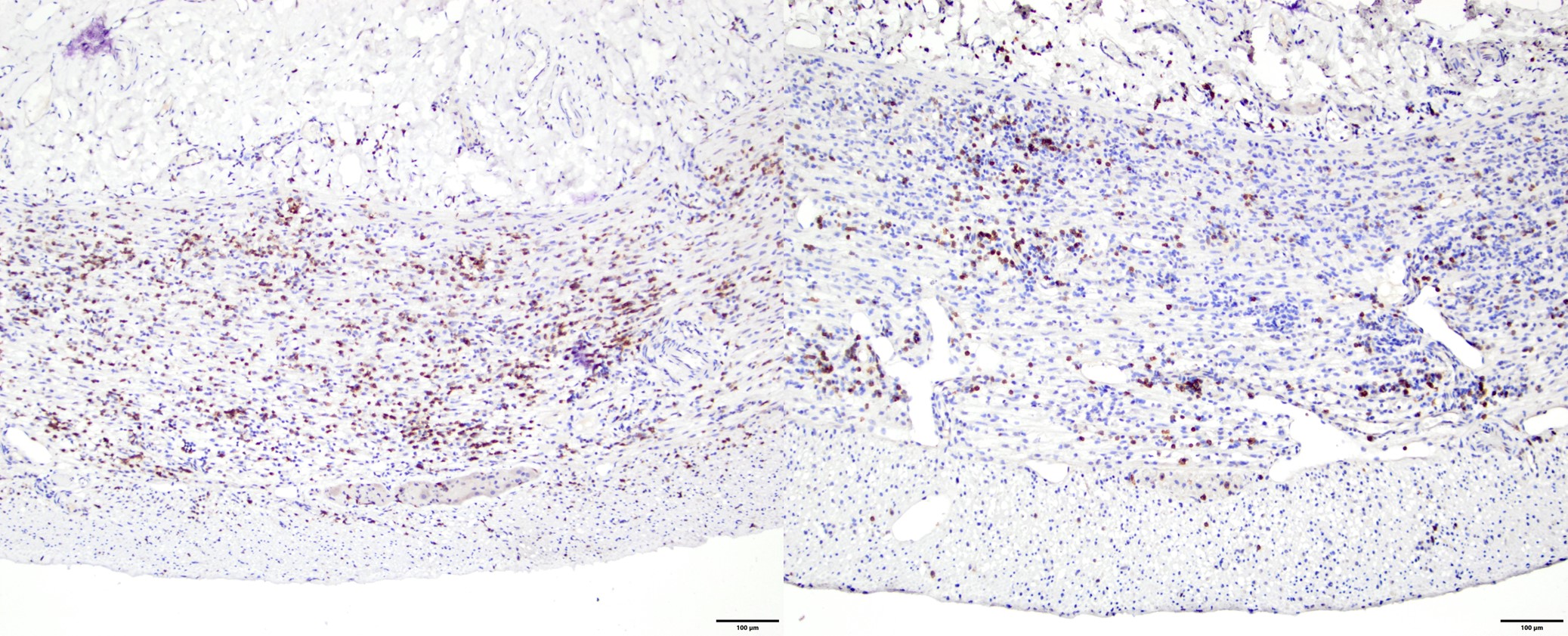

Supplement: Supplementary file 2 [file Image_2.jpeg]
